# Variation in Soil Microbial Communities Along an Elevational Gradient in Alpine Meadows of the Qilian Mountains, China

**DOI:** 10.3389/fmicb.2021.684386

**Published:** 2021-06-25

**Authors:** Yulong Duan, Jie Lian, Lilong Wang, Xuyang Wang, Yongqing Luo, Wanfu Wang, Fasi Wu, Jianhua Zhao, Yun Ding, Jun Ma, Yulin Li, Yuqiang Li

**Affiliations:** ^1^Northwest Institute of Eco-Environment and Resources, Chinese Academy of Sciences, Lanzhou, China; ^2^Naiman Desertification Research Station, Northwest Institute of Eco-Environment and Resources, Chinese Academy of Sciences, Tongliao, China; ^3^University of Chinese Academy of Sciences, Beijing, China; ^4^National Research Center for Conservation of Ancient Wall Paintings and Earthen Sites, Dunhuang Academy, Dunhuang, China; ^5^MOE Key Laboratory of Cell Activities and Stress Adaptations, School of Life Sciences, Lanzhou University, Lanzhou, China; ^6^Shanghai Majorbio Bio-Pharm Technology Co., Ltd., Shanghai, China; ^7^Gansu Qilian Mountains National Nature Reserve Authority, Zhangye, China

**Keywords:** alpine meadow ecosystem, biogeographic patterns, Qilian Mountains, soil microbial diversity, co-occurrence network, amplicon sequencing

## Abstract

Bacterial, archaeal, and eukaryota diversity in mountainous areas varies along elevational gradients, but details remain unclear. Here, we use a next-generation sequencing method based on 16S/18S rRNA to reveal the soil microbial diversity and community compositions of alpine meadow ecosystems along an elevation span of nearly 2,000 m (1,936–3,896 m) in China’s Qilian Mountains. Both bacterial and eukaryota diversity increased linearly with increasing elevation, whereas archaeal diversity increased, but not significantly. The diversity patterns of several phyla in the bacterial, archaeal, and eukaryota communities were consistent with the overall elevational trend, but some phyla did not follow this pattern. The soil microbial community compositions were shaped by the coupled effects of regional climate and local soil properties. Intradomain links were more important than interdomain links in the microbial network of the alpine meadows, and these links were mostly positive. The bacteria formed more connections than either archaea or eukaryota, but archaea may be more important than bacteria in building the soil microbial co-occurrence network in this region. Our results provide new visions on the formation and maintenance of soil microbial diversity along an elevational gradient and have implications for microbial responses to climate change in alpine ecosystems.

## Introduction

Studies of elevation clines in the soil biodiversity and compositions of ecological communities have provided important insights on the development of the fundamental theory of species diversity (Lomolino, 2001). Many studies that have been done over the years concluded that elevation influences the whole community’s structure through differential influences on their component species. These effects can either directly reflect the physiological responses of temperature and its related abiotic factors with elevation or indirectly reflect the effects of temperature on resources or symbiotic organisms (Sundqvist et al., 2013). These insights have enhanced research on the distribution patterns of animals, plants, and insects across continental ecosystems, which helped to explain general patterns along elevational gradients (Kessler et al., 2011; Descombes et al., 2017; Dvorsky et al., 2017).

However, soil microbes remain still partially neglected. For decades, studies have shown that the abundance and diversity of soil microbial communities varied along elevational gradients, but that the microbes do not follow the same patterns as animals or plants (Fierer et al., 2011). In particular, studies of bacterial diversity along elevational gradients have shown inconsistent trends, which can be classified into three main categories: (1) decreasing diversity with increasing elevation. For instance, one of the earliest studies reported that soil *Acidobacteria* diversity decreased from the lowest to the highest elevations in the American Rocky Mountains (Bryant et al., 2008). However, this study only examined *Acidobacteria* and did not represent the whole bacterial community. (2) Hump- or U-shaped patterns, with plant diversity gradually decreasing along an elevational gradient on Japan’s Mount Fuji and the soil bacterial diversity presenting a single peak diversity, whereas the archaea showed a double peak pattern (Singh et al., 2012). In addition, a U-shaped pattern was reported for soil bacterial diversity across an elevational gradient on Tanzania’s Mt. Kilimanjaro (Peay et al., 2017). (3) Independent trends. For example, research in the eastern Andes of Peru, the Changbai Mountain in China, and Mt. Halla in South Korea all showed that soil bacterial diversity did not change with elevation (Fierer et al., 2011; Shen et al., 2013; Singh et al., 2014).

Most studies of soil microbial diversity distribution patterns in mountain ecosystems focused on bacteria, whereas studies of soil fungal and archaeal communities remain limited, and the observed trends need further explanation. Because there is still great uncertainty about the factors that explain differences in the microbial elevational distribution across studies, these differences may result from the combined effects of multiple simultaneously varying environmental parameters or undetected factors associated with soil microbes, and the possibility that microbial diversity may not change at a spatial scale that corresponds to an elevational gradient (Shen et al., 2020).

Different microbial taxa or groups at multiple taxonomic levels may present distinct elevation patterns. For example, research on Mt. Kilimanjaro in East Africa showed a U-shaped bacteria diversity distribution pattern and a monotonically fungal decreasing trend (Shen et al., 2020). Similarly, soil bacterial diversity patterns across an elevational gradient on the Kohala volcano of Hawai’i showed a hump-shaped diversity distribution pattern versus a monotonically increasing pattern for fungi (Peay et al., 2017). In addition, studies in the Changbai Mountains displayed a linearly decreasing diversity pattern for bacteria, but an increasing pattern for fungi (Shen et al., 2015; Ni et al., 2018). A study of bacterial communities on Mt. Fuji found that the *Acidobacteria* diversity decreased with increasing elevation, whereas *Proteobacteria* showed a hump-shaped diversity pattern, and actinobacterial diversity showed no significant pattern (Singh et al., 2012).

α Diversity and composition of soil microbes along elevational gradients are affected by multiple environmental factors, and these factors can be summarized into two scale-dependent categories: (1) regional climate conditions, and particularly precipitation and temperature (Singh et al., 2014), and (2) local soil conditions such as the soil pH and nutrient content and the vegetation type (Shen et al., 2013, 2015; Zhang et al., 2013; Lin et al., 2015). However, the relative importance of regional climate conditions and local soil conditions on the soil microbial community’s elevational distribution patterns remains controversial. Some authors suggest that temperature strongly affects soil microbial diversity and community composition along an elevational gradients (Singh et al., 2014; Nottingham et al., 2018), whereas others suggest that soil pH is the most important driver of the elevational distribution (Shen et al., 2013, 2019; Wang et al., 2015; Peay et al., 2017). These differences can likely be attributed to differences between ecosystem types, elevations, research scales, microbial groups included in the study, and research techniques. In general, although previous studies of the soil microbial elevational diversity patterns have attracted considerable attention, the generality of these patterns and their underlying drivers are still poorly understood (Shen et al., 2019).

It is well known that microbes do not exist in isolation within a microbial community, and that they participate in complicated interaction systems that determine the microbial community compositions (Freilich et al., 2010). Microbes coexist and develop complex networks through positive interactions such as mutualism, negative interactions such as competition, and neutral interactions such as commensalism (Faust and Raes, 2012). Previously, most analytical methods of soil microbial communities focused on microbial diversity, community compositions, and their response to environmental factors, but did not examine the relationships among soil microbial species (Deng et al., 2012). In the past decade, network analysis has been demonstrated that it gives us new insights into microbial community ecology that cannot be obtained using traditional analytical approaches (Barberán et al., 2012; Faust and Raes, 2012; Li and Wu, 2018; Shi et al., 2019; Tu et al., 2020).

The Qinghai–Tibet Plateau is known as “the earth’s third pole.” Moreover, it was one of the most sensitive regions to global climate change (Qiu, 2008). In this region, alpine grassland (including alpine meadow and alpine steppe) is the most important vegetation type and occupies more than 60% of the total area and plays an important role in maintaining the biodiversity of the alpine ecosystem (Ding et al., 2013). In recent years, many studies of soil microorganism at sample-plot scale and sample-transect scale have been carried out in the Qinghai–Tibet Plateau, including its spatial–temporal distribution patterns, driving factors, and responses to global change and human activities (Li et al., 2016; Shi et al., 2016, 2019; Zhang et al., 2016; Yang et al., 2017; Shen et al., 2019). The Qilian Mountains are on the northern boundary of the Qinghai–Tibet plateau. They extend more than 1,000 km from east to west with an average elevation of 4,000 to 5,000 m. The mountains form an important ecological security barrier in Western China. The mountains protect the oases in the Hexi Corridor and the Qinghai–Tibet Plateau from encroachment by the Tengger Desert and Badain Jaran Desert. The mountains are also an important source basin for the Yellow River and a priority area for biodiversity protection in China. However, as an indispensable part of the Qinghai–Tibet Plateau, the detailed soil microbial ecology of this region has been poorly investigated. Our goal was to provide some of the missing information. Specifically, our goals were to (1) investigate the community diversity, species richness, and composition of three soil microbial domains (bacteria, archaea, and eukaryota) along an elevational gradient in alpine meadows of the Qilian Mountains; (2) map the elevational distribution of the soil microbial community in this region and determine the main driving factors; and (3) reveal the soil microbial co-occurrence patterns and relative importance of bacteria, archaea, and eukaryota in building the microbial co-occurrence network. Hence, our study could help to not only enhance our entire understanding of the soil microbial ecology in the Qinghai–Tibet Plateau, but also formulate the management strategies of the alpine ecosystem in answering climate change.

## Materials and Methods

### Study Site and Soil Sampling

Soil samples were collected at 14 sites over a broad area of alpine meadows in the northern foothills of the Qilian Mountains in August 2019 (Supplementary Table 1). At each site, the surface soil to a depth of 20 cm was collected at five random locations within a square plot (10 m × 10 m) and homogenized to produce a single composite soil sample. The samples were packed in sterilized polyethylene sample tubes and transported to the laboratory in portable car refrigerators rapidly. Soil samples were then divided into two subsamples: one was stored at 4°C to determine the soil properties, and the other was stored at −80°C prior to DNA extraction.

### Environmental Data and Soil Physicochemical Properties

The environmental parameters were geographic variables (latitude, longitude, and elevation) and meteorological data [mean annual precipitation (MAP) and mean annual temperature (MAT)] at each sampling site and soil properties [pH, electrical conductivity (EC), total carbon (TC), total nitrogen (TN), and the TC/TN ratio] for each sample. The meteorological data (MAP and MAT) were collected from the Chinese Meteorological Agency Database^1^. Soil pH was measured by using an E20-FiveEasy pH meter (Mettler Toledo, Giessen, Germany), and the soil EC was calculated by using an electric conductometer. Both soil measurements used a soil water suspension (a 5:1 vol/vol mixture of deionized water and soil) after shaking for 30 min. Soil TC and TN were determined with a carbon–hydrogen–nitrogen elemental analyzer (2400 II CHN Elemental Analyzer; PerkinElmer, Boston, MA, United States). Meteorological data and soil properties are shown in Supplementary Tables 1, 2, respectively.

### Soil DNA Extraction and MiSeq High-Throughput Sequencing

Soil DNA in each site was extracted from approximately 0.5 g of soil under sterile conditions by using the Fast DNA SPIN Kit for Soil (MP Biomedicals, Solon, OH, United States) according to the manufacturer’s instructions. We used 1.0% agarose gel electrophoresis and a NanoDrop ND-2000 spectrophotometer (Thermo Scientific Inc., Waltham, MA, United States) to measure the DNA yield and quality. DNA samples were kept at −80°C until use. Here, we amplified the bacterial 16S rRNA V3–V4 hypervariable regions, archaeal 16S rRNA V3–V5 hypervariable regions, and eukaryota 18S rRNA V5–V7 hypervariable regions by the universal primer sets of 338F/806R (Xu et al., 2016), Arch344F/Arch915R (Ohene-Adjei et al., 2007), and 817F/1196R (Rousk et al., 2010), respectively.

Polymerase chain reaction (PCR) volume (20 μL) contained 5 × reaction buffer (4 μL), dNTPs (4 μL of 2.5 mM), each primer (0.8 μL of 5 μM), template DNA (1 μL of approximately 10 ng), and Pfu DNA polymerase (0.4 μL) (TransGen Biotech, Beijing, China), with ultrapure H_2_O to make up to the final volume. The PCR conditions for bacteria are as follows: 95°C for 3 min, 27 cycles at 95°C for 30 s, 55°C for 30 s, and 72°C for 45 s with a final extension at 72°C for 10 min. The PCR conditions for archaea and eukaryota are as follows: 95°C for 3 min, 35 cycles at 95°C for 30 s, 55°C for 30 s, and 72°C for 45 s with a final extension at 72°C for 10 min. High-throughput sequencing was performed on an Illumina MiSeq platform (Illumina, San Diego, CA, United States) and 300-bp paired-end reads were generated in Majorbio Co., Ltd. (Shanghai, China). All sequencing data were deposited to the NCBI Sequence Read Archive^2^ under project number PRJNA676004.

### Bioinformatics and Statistical Analyses

Forward and reverse paired-end sequences from MiSeq platform were merged using FLASH (v. 1.2.7)^3^ (Magoč and Salzberg, 2011). The paired-end reads were assigned to each sample based on the unique barcode sequence and then analyzed using QIIME (v. 1.9.0) (Caporaso et al., 2010). The raw FASTQ files were quality-filtered using fastp (v.0.20.0)^4^ (Chen et al., 2018) with the following criteria: reads shorter than 300 bp or *Q* < 20 were discarded. Briefly, quality-filtered sequences were aligned in accordance with SILVA alignment (Quast et al., 2013) and then clustered into operational taxonomic units (OTUs) with 97% sequence similarity threshold using UPARSE (v. 7.1)^5^ (Edgar, 2013). To minimize the effects of sequencing depth on the α- and β-diversity measures, we rarefied the numbers of bacterial, archaeal, and eukaryota sequences from each sample to 30,671, 17,561, and 42,299, respectively, which yielded an average Good’s coverage of 98.0, 99.4, and 100.0%, respectively. We calculated α-diversity indexes for richness estimators (ACE and Chao1) and diversity indices (Shannon and Simpson) and calculated the coverage using MOTHUR (v. 1.30.2)^6^ (Schloss et al., 2009). Taxonomic annotation of the OTUs was performed using MOTHUR in accordance with SILVA (132)^7^ or Unite (8.0)^8^, with 70% confidence intervals. We used principal components analysis (PCA) to show the difference in β-diversity between samples along the elevational gradient and visualized the results using the ggplot2 package in R (v. 3.6.1)^9^. We used redundancy analysis (RDA) to show the effects of the environmental factors on driving the microbial communities’ differentiation, with elevation, longitude, MAP, MAT, soil pH, TN, TC, the TC/TN ratio, and EC as the explanatory factors. Subsequently, we applied variance partitioning analysis (VPA) to quantitatively evaluate the individual and group contributions to the total variance between environmental factors and soil microbial communities using the vegan package for the R software^10^. We inferred the soil microbial co-occurrence network by using sparse correlations for compositional (Friedman and Alm, 2012) correlation matrix constructed using the weighted correlation network analysis package in R (3.6.1) (Langfelder and Horvath, 2012). To eliminate background interference from rare OTUs, we discarded OTUs with a relative abundance less than or equal to 0.05% of the total number of bacterial, archaeal, and eukaryota sequences from the data pool. The network nodes thus represent OTUs, and the edges connecting these nodes represent correlations between OTUs (Shi et al., 2019). The thickness of each edge is proportional to the magnitude of the correlation coefficient (Spearman *r* > ± 0.6 and *P* < 0.01).

## Results

### Microbial Diversity Characterization and Community Composition in the Alpine Meadows of the Qilian Mountains

**FIGURE 1:**
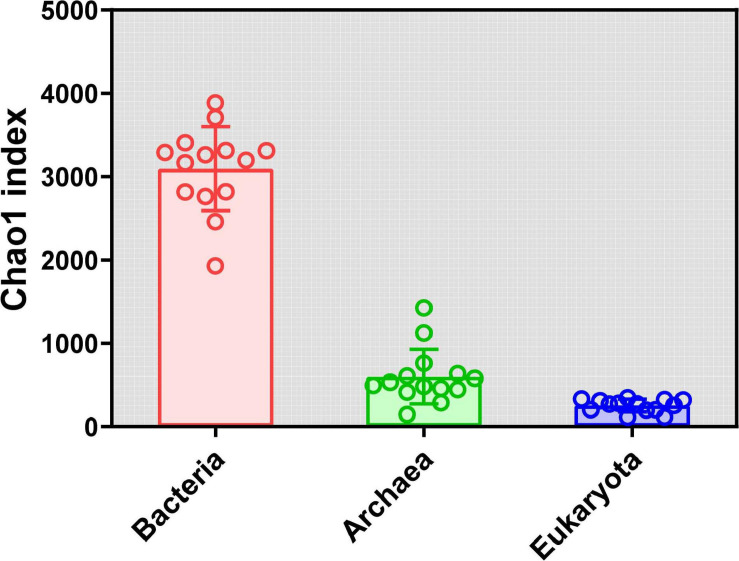
α Diversity (Chao1 index) analysis for the soil microbial community in alpine meadows in the Qilian Mountains.

**FIGURE 2:**
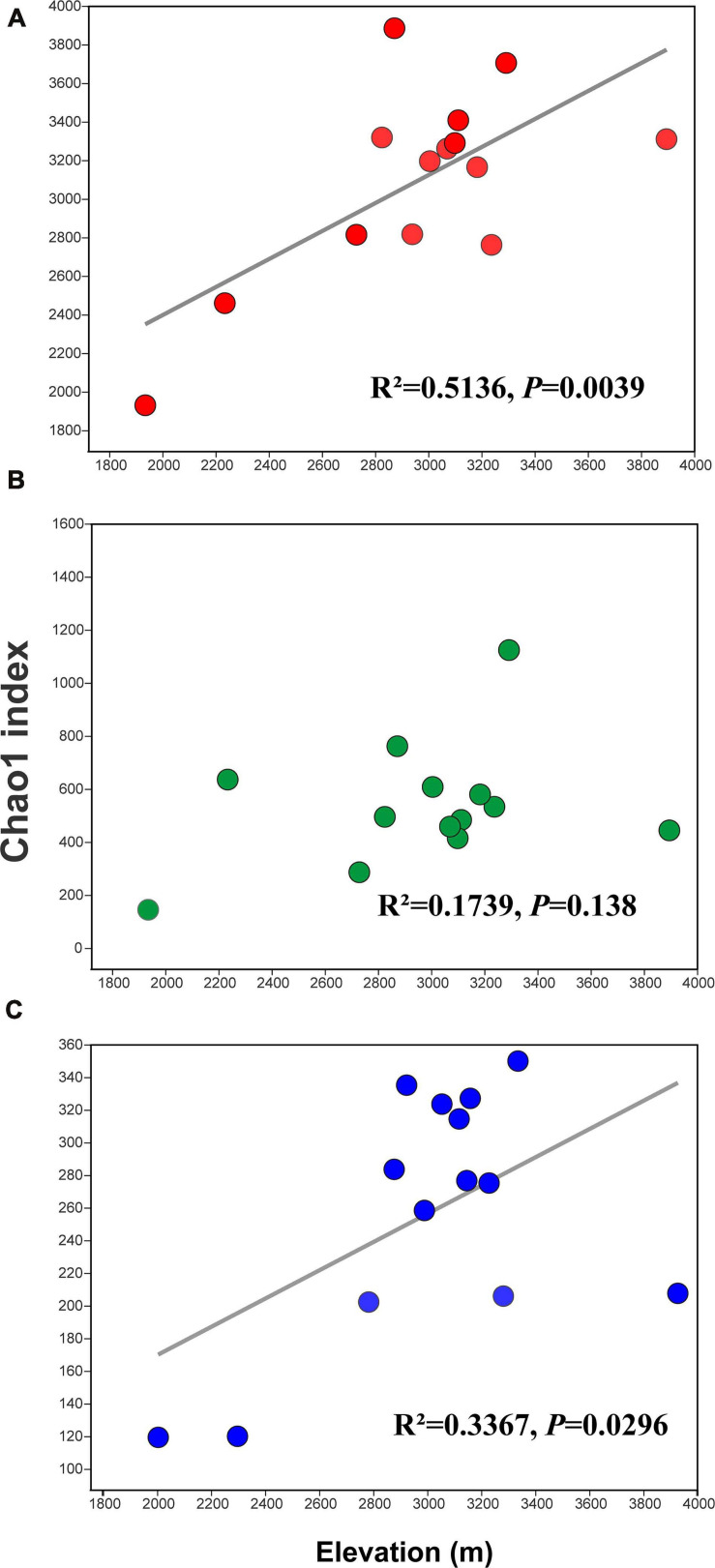
Linear regression analysis for the relationships between the Chao1 index and elevation. Values are for **(A)** the bacterial samples, **(B)** the archaeal samples, and **(C)** the eukaryota samples.

Rarefaction curves of all samples in our study indicate that sequenced reads in samples met satisfactory standards (Supplementary Figure 1). α Diversity was estimated using the Chao1 index. For the bacteria, the Chao1 index ranged from 1,929.46 to 3,884.24, and the mean and median were 3,093.10 and 3,227.56, respectively (Supplementary Table 3). For the archaea, the Chao1 index ranged from 144.11 to 1,423.79, and the mean and median were 598.79 and 513.42, respectively (Supplementary Table 4). For the eukaryota, the Chao1 index ranged from 113.00 to 346.25, and the mean and median were 252.32 and 271.39, respectively (Supplementary Table 5). Clearly, the Chao1 index of the soil bacterial community was far higher than those for the archaeal community and eukaryota communities (Figure 1). The number of observed OTUs, ACE, Shannon, and Simpson indices for the bacteria, archaea, and eukaryota are summarized in Supplementary Tables 3–5, respectively. Furthermore, we found different diversity patterns in bacterial, archaeal, and eukaryotic communities along the elevational gradient. The Chao1 index of the soil bacterial community (*R*^2^ = 0.5136, *P* = 0.0039) (Figure 2A) and the eukaryota community (*R*^2^ = 0.3367, *P* = 0.0296) (Figure 2C) increased significantly with increasing elevation. Meanwhile, the Chao1 index of soil archaeal community showed no significant correlation with elevation (Figure 2B).

For the α-diversity of the top 10 bacterial phyla, five of the phyla showed a statistically significant (*P* < 0.05) increase with increasing elevation: *Acidobacteria*, *Bacteroidetes*, *Patescibacteria*, *Proteobacteria*, and *Verrucomicrobia* (Supplementary Figure 2). For the α-diversity of the top five archaeal phyla, only one phylum showed a statistically significant (*P* < 0.05) relationship with elevation: a significantly decreasing diversity for *Thermoplasmatota* (Supplementary Figure 3). For the α-diversity of the top five eukaryota phyla, two showed significantly (*P* < 0.05) increasing diversity with increasing elevation (SAR_norank and Fungi_unclassified) and one (*Mucoromycota*) showed a marginally significant increase (*P* = 0.064) (Supplementary Figure 4).

**FIGURE 3:**
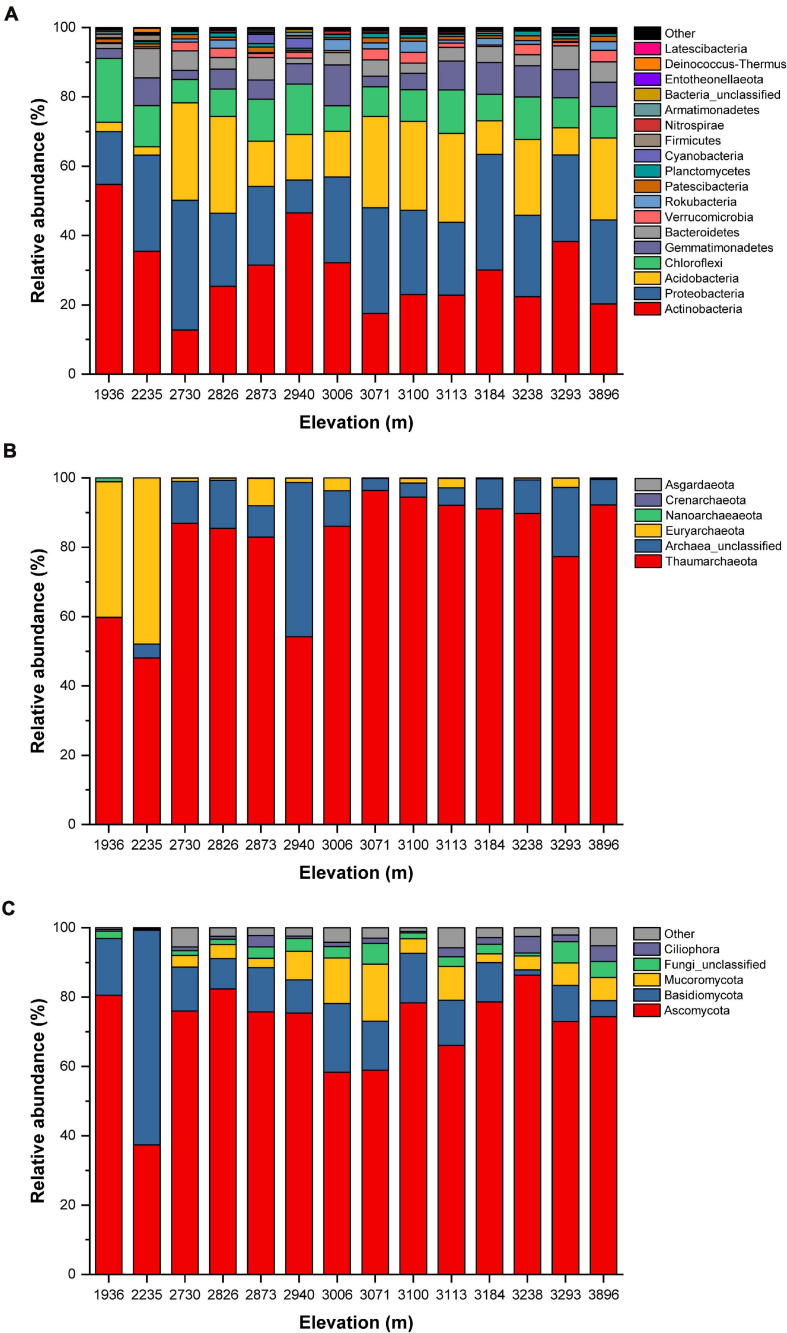
Relative abundance (%) of the **(A)** bacterial, **(B)** archaeal, and **(C)** eukaryota communities at the phylum level.

We obtained a total of 684,348 valid sequences and 8,001 OTUs from the 14 sample sites. Each sample contained from 35,829 to 58,887 sequences, representing 1,763 to 2,921 phylogenetic OTUs (Supplementary Table 3). All sequences could be assigned into 36 different bacterial phyla. Of these, nine dominated the bacterial community: *Actinobacteria* (12.7–54.8%), *Proteobacteria* (9.4–37.5%), *Acidobacteria* (2.4–28.1%), *Chloroflexi* (6.8–18.4%), *Gemmatimonadetes* (2.6–11.8%), *Bacteroidetes* (1.4–8.5%), *Verrucomicrobia* (0.2–3.2%), *Rokubacteria* (<0.1%–3.2%), and *Patescibacteria* (0.4–1.6%). These phyla accounted for 93.4% to 98.0% of the overall bacterial library; the remaining phyla, which each accounted for less than 1% of the total, comprised only 2.0 to 6.6% of the whole bacterial community (Figure 3A and Supplementary Table 6). For the archaea, we obtained 462,259 valid sequences, representing a total of 2,173 OTUs, with each sample containing from 21,266 to 48,950 valid sequences and 129 to 1,172 phylogenetic OTUs (Supplementary Table 4). Only six archaeal phyla were detected in the soils. *Thaumarchaeota* was the dominant phylum at all elevations (48.1–96.4%), followed by *Euryarchaeota* (<0.1–47.9%) and Archaea_unclassified (<0.1–44.5%). These three phyla accounted for 99.0 to 100.0% of the overall archaeal library; the other phyla accounted for 1% or less of the total (Figure 3B and Supplementary Table 7). For the eukaryota, we obtained 870,813 valid sequences and 644 OTUs, with each library containing from 45,895 to 73,633 sequences and 92 to 323 phylogenetic OTUs (Supplementary Table 5). A total of 36 eukaryota phyla were detected across all soils. *Ascomycota* (37.4–86.3%) was the dominant eukaryota phylum at all elevations, across all soil samples, followed by *Basidiomycota* (1.6–62.0%), *Mucoromycota* (< 0.1–16.4%), Fungi_unclassified (0.4–6.2%), and *Ciliophora* (0.1–4.8%). Of these phyla, *Ascomycota*, *Basidiomycota*, *Mucoromycota*, and Fungi_unclassified belonged to the fungi and accounted for 90.3 to 99.7% of the overall eukaryota library. However, there was a significant number of non-fungal eukaryota sequences, including *Ciliophora*, which are invertebrates. The other phyla represented a very small fraction (0.2–5.7%) of the total eukaryota community (Figure 3C and Supplementary Table 8).

### Correlations Between Soil Variables, Soil Microbial Diversity, and Community Composition

Linear regression analysis indicated the Chao1 index for the bacterial community was significantly negatively correlated with the soil TC/TN ratio (*R*^2^ = 0.5382, *P* = 0.0028) and significantly positively correlated with TN (*R*^2^ = 0.2899, *P* = 0.047), but not significantly correlated with the other soil parameters (Table 1 and Supplementary Table 9). For the archaea, the Chao1 index for the soil archaeal community was significantly negatively correlated with the soil TC/TN ratio (*R*^2^ = 0.3283, *P* = 0.0322) (Table 1 and Supplementary Table 9). For the eukaryota, the Chao1 index was significantly positively correlated with MAP (*R*^2^ = 0.3854, *P* = 0.0178) and TN (*R*^2^ = 0.3200, *P* = 0.035) and significantly negatively correlated with the TC/TN ratio (*R*^2^ = 0.6137, *P* = 0.0009) (Table 1 and Supplementary Table 9). Obviously, soil TC/TN ratio provided the greatest contribution to explaining the α-diversity.

**TABLE 1:** Results of the linear regression analysis for the relationships between the Chao1 index for the three groups of soil microbes and the environmental variables.

Chao 1	Bacteria			Archaea			Eukaryota		
	Equation	*R*^2^	*P*	Equation	*R*^2^	*P*	Equation	*R*^2^	*P*
Latitude	*y* = –152.814*x* + 8,320.053	0.0700	0.3605	*y* = −71.890*x* + 3,200.752	0.0884	0.3018	*y* = −33.762*x* + 1,465.446	**0.3699**	0.021*
Longitude	*y* = 24.5453*x* − 47.399	0.0055	0.8019	*y* = 24.721*x*–2,039.069	0.0293	0.5582	*y* = 14.565*x–*1,287.760	0.2077	0.1015
Elevation	*y* = 0.6097*x* + 576.3409	**0.5136**	0.0039**	*y* = 0.154*x*−48.942	0.1739	0.138	*y* = 0.047*x* + 12.837	**0.3367**	0.0296*
MAT	*y* = −21.143*x* + 2,282.396	0.2492	0.0692	*y* = −0.274*x* + 405.570	0.0002	0.9597	*y* = −1.292*x* + 147.293	0.1007	0.2689
MAP	*y* = 0.815*x* + 2,154.836	0.0781	0.3333	*y* = 0.312*x* + 320.265	0.0606	0.3961	*y* = 0.174*x* + 104.982	**0.3854**	0.0178*
pH	*y* = 251.013*x* + 232.281	0.0256	0.585	*y* = −90.691*x* + 1,183.246	0.0177	0.6501	*y* = −8.639*x* + 227.289	0.0033	0.8459
TC	*y* = 135.052*x* + 1,937.780	0.2091	0.1002	*y* = 44.468*x* + 260.860	0.1203	0.2244	*y* = 11.129*x* + 116.791	0.1536	0.1658
TN	*y* = 1,678.359*x* + 1,999.04	**0.2899**	0.047*	*y* = 586.964*x* + 273.213	0.1882	0.1212	*y* = 169.522*x* + 114.734	**0.3200**	0.035*
TC/TN ratio	*y* = −17.451*x* + 2,732.161	**0.5382**	0.0028**	*y* = −5.916*x* + 525.830	**0.3283**	0.0322*	*y* = −1.791*x* + 189.362	**0.6137**	0.0009***
EC	*y* = 0.028*x* + 2,367.895	0.0016	0.892	*y* = 0.087*x* + 365.784	0.0813	0.3231	*y* = −0.002*x* + 154.27	0.0009	0.9197

MAT, mean annual temperature; MAP, mean annual precipitation; EC, electrical conductivity; TC, total carbon; TN, total nitrogen. Correlations with significant values (*P < 0.05, **P < 0.01, ***P < 0.001) are shown in bold.

**FIGURE 4:**
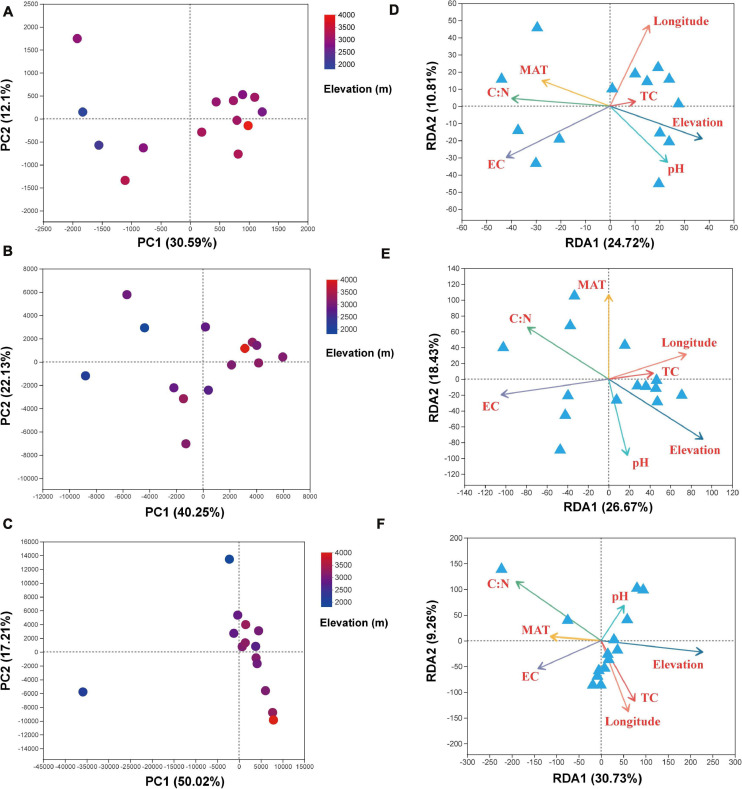
Results of the principal components (PC1 and PC2) analysis for the soil **(A)** bacterial, **(B)** archaeal, and **(C)** eukaryota communities and redundancy analysis results (RDA1 and RDA2) for the soil **(D)** bacterial, **(E)** archaeal, and **(F)** eukaryota community diversity and the environmental variables. Variables: MAT, mean annual temperature; MAP, mean annual precipitation; EC, electrical conductivity; TC, total carbon; TN, total nitrogen.

The PCA analysis at the OTU level showed the β-diversity of bacterial, archaeal, and eukaryota community (Figures 4A–C). Principal component (PC) 1 explains the majority of the variance in our data and elevation (sampling location) maps well onto this PC axis. For the bacteria, soil samples form a gradient from left side to right side along PC1, with 30.6% of the total variation, ranging from low elevations to high elevations, whereas samples from low elevations were separate from the other samples along PC2, with 12.1% of the total variation (Figure 4A). For the archaea, soil samples also present a gradient from left to right along PC1, with 40.2% of the total variation, ranging from low elevations to high elevations, whereas samples from low elevations were separated from the other samples along PC2, with 22.1% of the total variation (Figure 4B). For the eukaryota, samples from high elevations were gathered together along PC1, with 50.0% of the total variation, whereas samples from low elevations were separated from the other samples along PC2, with 17.2% of the total variation (Figure 4C). Subsequently, the RDA was used to shed light on the influence of variation of soil physicochemical characteristics on soil microbial community composition (Figures 4D–F). For the bacteria (Figure 4D), RDA1 and RDA2 explained 24.7 and 10.8% of the community compositions, respectively. Among the all-environmental factors we measured, EC and TC/TN ratio of the soils were the dominant factors responsible for promoting the changes of soil microbial community compositions along the RDA1, whereas the longitude of the sampling location was the primary factor contributing to shifting the communities along the RDA2 (Supplementary Table 10). For the archaea (Figure 4E), RDA1 and RDA2 explained 26.7 and 18.4% of the archaeal community compositions, respectively. Among the environmental factors, EC was the primary factor contributing to shifting the community compositions along the RDA1, whereas MAT was the dominant driver for the community variation along the RDA2 (Supplementary Table 10). For the eukaryota (Figure 4F), RDA1 and RDA2 explained 30.7 and 9.3% of the eukaryota community compositions variation, respectively. Among the environmental factors, elevation was the dominant driver for changing of community compositions along the RDA1, whereas the longitude of the sampling location was the dominant factor driving the changes of communities along the RDA2 (Supplementary Table 10).

**FIGURE 5:**
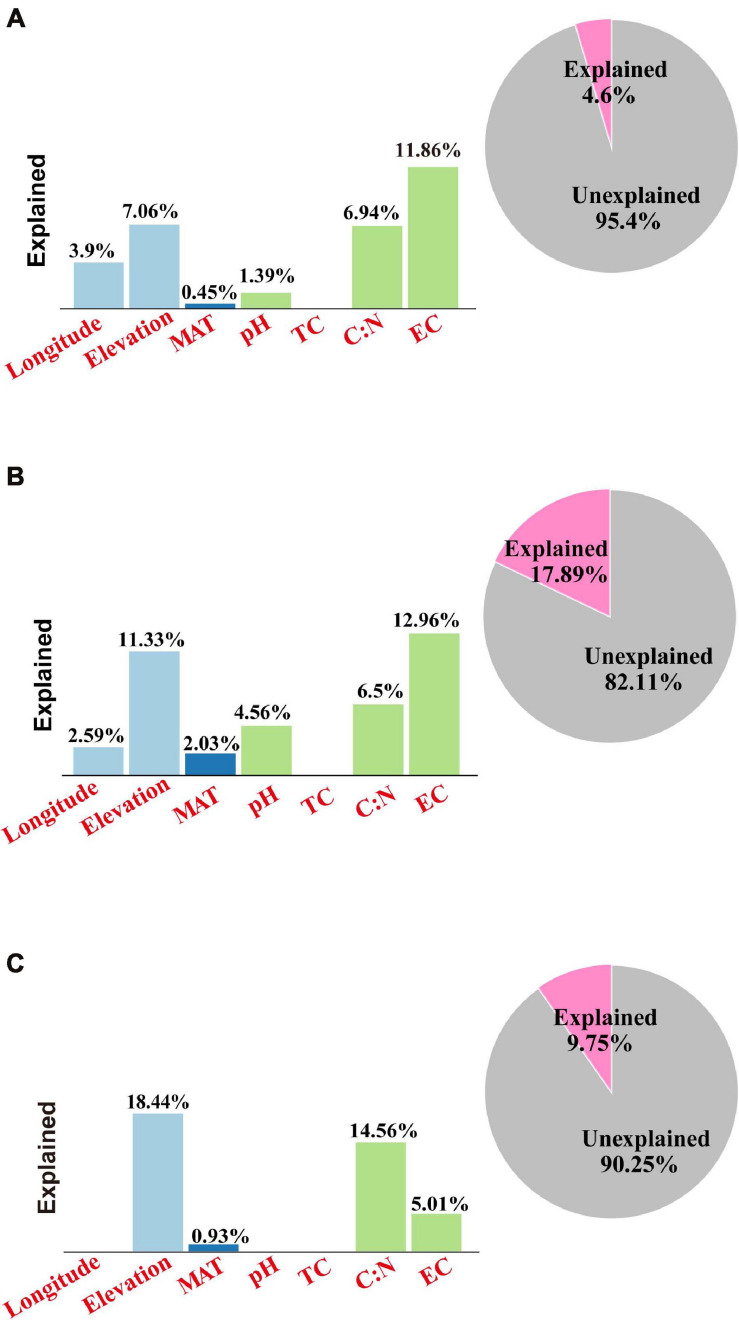
Variation partition analysis (VPA) results for the environmental variables for the soil **(A)** bacterial, **(B)** archaeal, and **(C)** eukaryota communities along the elevational gradient in alpine meadows of the Qilian Mountains. Variables: MAT, mean annual temperature; MAP, mean annual precipitation; EC, electrical conductivity; TC, total carbon; TN, total nitrogen. The “Explained” in bar graphs represent the individual explanation of single environmental variables, whereas the “Explained” in pie graphs represent the whole explanation of all environmental variables.

Finally, VPA was applied in the quantitative evaluation of the individual and common degree of interpretation between variants of environmental factors and soil microbial community composition (Figure 5). For the bacteria, VPA showed that the elevation explained 7.1% of the community variation, and the selected soil parameters explained 20.2% of the variation, with soil EC explaining 11.9% of the community variation and the soil TC/TN ratio explaining 6.9% of the community variation, leaving 95.4% of the unexplained variation (Figure 5A). For the archaea, VPA showed that the elevation explained 11.3%, with the selected soil parameters explaining 24.0% of the variation, EC explaining 13.0% of the total community variation, and the soil TC/TN ratio explaining 6.5% of the community variation, leaving 82.1% of the unexplained variation (Figure 5B). For the eukaryota, VPA showed that the elevation explained 18.4% of the community variation, and the selected soil parameters explained 19.6% of the variation, with soil TC/TN ratio being the master variable by explaining 14.6% of the community variation, leaving 90.2% of the variation unexplained (Figure 5C).

### Soil Microbial Network Analysis

**FIGURE 6:**
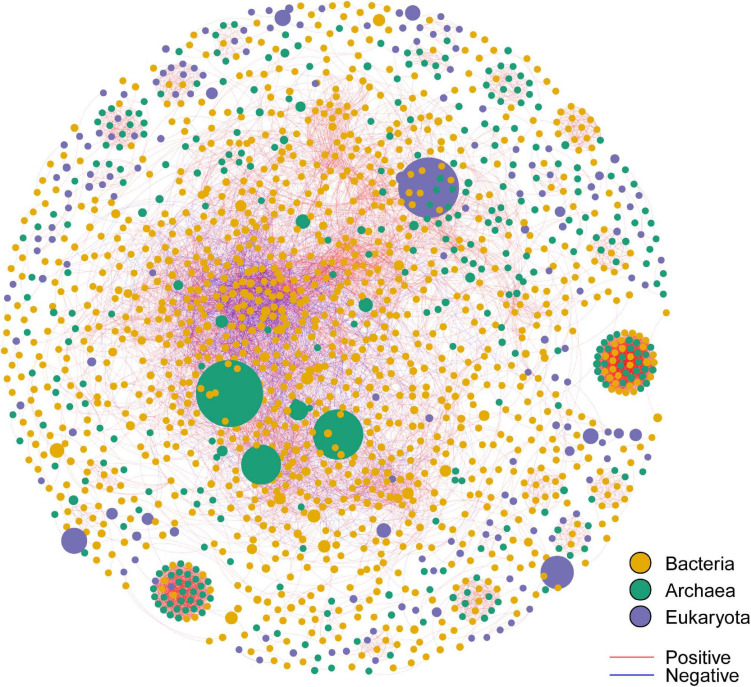
The co-occurrence network for the bacterial OTUs, archaeal OTUs, and eukaryota OTUs in the alpine meadow’s soils of the Qilian Mountains. The size of each node is proportional to the relative abundance of the OTU; nodes with the same color belong to the same domain; the links in red color represent positive interaction, and those in blue represent negative interaction. OTUs with a relative abundance of less than 0.05% were eliminated. The thickness of each edge (i.e., each connection between nodes) is proportional to the magnitude of the correlation coefficient (Spearman *r* > ± 0.6 and *P* < 0.01).

**FIGURE 7:**
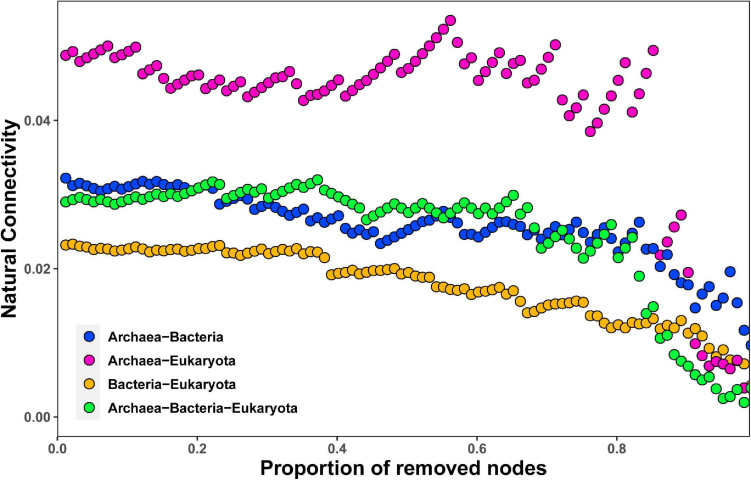
Network robustness analysis of containing archaea–bacteria, archaea–eukaryota, bacteria–eukaryota, and archaea–bacteria–eukaryota microbial groups in the alpine meadow’s soils of the Qilian Mountains.

By employing co-occurrence network analyses to the bacterial, archaeal, and eukaryota sequence data, we built a microbial network for the alpine meadows of the Qilian Mountains (Figure 6). The network contained 1,701 nodes (i.e., taxa) and 11,283 edges that connected them (Table 2). Among them, the edges for bacteria in the network included 5,367 positive correlations and 1,677 negative correlations in the samples; for the archaea, 1,429 edges in the network included 1,428 positive correlations and only one negative correlation; and for the eukaryota, 139 edges in the network included 139 positive correlations and no negative correlations (Table 2). In addition, there were more intradomain links than interdomain links (Supplementary Table 11), and most of these links were positive (Table 2 and Supplementary Table 11). In particular, the bacteria (9,463 links) formed more connections than either the archaea (1,681 links) or the eukaryota (139 links) (Supplementary Table 11). To reveal the relative importance of soil bacteria, archaea, and eukaryota in building the microbial co-occurrence network, we calculated the correlation frequency (number of real links/numbers of possible links) among the bacteria, archaea, and eukaryota. With the archaea absent, the correlation frequency between bacteria and eukaryota (1.048) was slightly lower than that between bacteria and archaea with eukaryota absent (1.160), but far lower than that between archaea and eukaryota with bacteria absent (1.728) (Table 3). Furthermore, the network robustness (as expressed by natural connectivity) analysis was conducted. Although natural connectivity gradually decreased as more nodes of OTUs were removed, the connectivity values generated with the archaea absent were significantly lower than those with bacteria or eukaryota omitted (Figure 7).

**TABLE 2:** Topological features of the microbial network in the alpine meadow’s soils of the Qilian Mountains.

Topological characters	Bacteria	Archaea	Eukaryota	Total
No. of node	1,181	354	166	1,701
No. of edge	7,044	1,429	139	11,283
Density	0.0078	0.0101	0.0229	0.0101
Degree	13.2663	11.9289	8.0734	1.6747
Node betweenness centrality	1,326.94	971.5639	31.6441	0.1988
Transitivity	0.6279	0.4112	0.9635	0.9441
Positive links (%)	5,367 (76.19%)	1,428 (99.93%)	139 (100.00%)	6,934 (80.51%)
Negative links (%)	1,677 (23.81%)	1 (0.07%)	0 (0.00%)	1,678 (19.48%)

**TABLE 3:** The correlation frequency (no. of real links/no. of possible links) between the groups of bacteria, archaea, and eukaryota based on an analysis of their absence and presence in the alpine meadow’s soils of the Qilian Mountains.

		Correlation frequency			
Absence	Presence	(Real/possible)	Bacteria	Archaea	Eukaryota
		———————————–% ———————————–			
Bacteria	Archaea	1.728	0	2.929	0.552
	Eukaryota			0.552	1.295
Archaea	Bacteria	1.048	1.296	0	0.151
	Eukaryota		0.151		1.295
Eukaryota	Bacteria	1.160	1.296	0.670	0
	Archaea		0.670	2.929	

## Discussion

### Similar Elevational Diversity Patterns Were Observed for Bacteria, Archaea, and Eukaryota

The results show that the soil microbial diversity was positively associated with the elevational gradient in the alpine meadows of the Qilian Mountains. This trend was statistically significant for bacteria and eukaryota (*P* < 0.05), but not for archaeal diversity (Figure 2). This is probably mainly attributed to the archaea excellent adaptability to barren and hash environments [e.g., high elevation, low oxygen, strong ultraviolet (UV) light, and rapidly changing weather) formed in their long-term evolution, leading archaea to be insensitive to changes in the external environment (Shi et al., 2016; Eme et al., 2017). In previous studies, which occurred along elevational gradients or in areas with homogeneous environmental factors, soil microbial diversity tended to decrease with increasing elevation (Nottingham et al., 2018), formed a hump-shaped or U-shaped pattern (Peay et al., 2017; Shen et al., 2020), or formed an insistent trend pattern (Singh et al., 2014). Recently, studies on Mt. Kilimanjaro in East Africa and Kohala volcano in Hawai’i presented contrasting patterns of soil bacterial and fungal diversity across an elevational gradient (Peay et al., 2017; Shen et al., 2020). Latitudinal gradients emulate elevation gradients, as temperatures decrease with both increasing elevation and increasing latitude. For example, a global-scale study and a continental-scale study of soil microorganisms also exhibited contrasting or decoupled diversity patterns across a latitudinal gradient, with bacteria showing a hump-shaped diversity pattern with increasing latitude, but decreasing fungal diversity with increasing latitude (Bahram et al., 2018; Liu et al., 2020).

Therefore, our results differ from these previous studies, which showed a remarkable increasing diversity pattern of soil bacteria and eukaryota across an elevational gradient, although the increase was not significant for archaeal diversity. Furthermore, our in-depth analysis of soil microbial diversity patterns at the phylum taxonomic level showed inconsistent diversity patterns for certain phyla (Supplementary Figures 2–4). For example, in agreement with the trend for the whole bacterial community, *Acidobacteria*, *Bacteroidetes*, *Patescibacteria*, *Proteobacteria*, and *Verrucomicrobia* showed significantly increasing diversity with increasing elevation, whereas the diversity of *Actinobacteria*, *Chloroflexi*, *Gemmatimonadetes*, *Methylomirabilota*, and *Myxococcota* showed no significant increase (Supplementary Figure 2). Similarly, in the eukaryota, the diversity of SAR_norank and Fungi_unclassified followed the fungal community trend, with significantly increasing diversity with increasing elevation (Supplementary Figure 4). However, the overall archaeal community did not exhibit a significant relationship with elevation, but the phylum *Thermoplasmatota* showed significantly decreasing diversity with increasing elevation (Supplementary Figure 3). The relationships between the above individual taxa and elevation are likely to contribute to the collectively microbial community change with increasing elevation (Yeh et al., 2019). Overall, the selected microbial diversity patterns are shaped by niche differentiation among different taxonomic groups that develop along gradients (Fierer et al., 2007).

### Patterns of Soil Microbial Diversity and Community Compositions Along Elevation Were Shaped by Various Environmental Drivers

We found abundant bacterial and eukaryotic taxa and relatively low-abundance archaeal taxa in the alpine meadows of the Qilian Mountains. We found a total of 36 bacterial phyla, mainly including *Actinobacteria*, *Proteobacteria*, *Acidobacteria*, *Chloroflexi*, *Gemmatimonadetes*, and *Bacteroidetes* (Figure 3A and Supplementary Table 6), and 36 eukaryota phyla, including *Ascomycota*, *Basidiomycota*, *Mucoromycota*, Fungi_unclassified, and *Ciliophora* (Figure 3C and Supplementary Table 8). In addition, several archaeal phyla were also detected in this region, including *Thaumarchaeota*, Archaea*_*unclassified, and *Euryarchaeota* (Figure 3B and Supplementary Table 7). These phyla showed no site specificity and were widely distributed in various ecosystems, especially in mountains (Bryant et al., 2008; Singh et al., 2012, 2014, 2016; Bahram et al., 2018; Tu et al., 2020).

Elevation is nearly always a complex and indirect gradient, as various environmental factors change in complicated ways along the gradient (Fierer et al., 2011). Thus, soil microbial diversity along elevational gradients will be affected by the simultaneous effects of these environmental factors. For example, soil pH has generally been demonstrated to be a principal driving force for soil microbial diversity patterns along elevational gradients with different scales (Shen et al., 2013, 2019, 2020; Wang et al., 2015). Recently, the contribution of soil pH in shaping bacterial and eukaryotic community composition was also detected along elevational distributions (Yuan et al., 2014). Of course, pH is not the only important factor; other environmental factors, including regional climatic factors (MAP and MAT) and edaphic variables (TC, TN, TC/TN ratio, and EC) (Auguet et al., 2009; Bahram et al., 2018; Ni et al., 2018; Nottingham et al., 2018), are significantly correlated with elevation and would have contributed to the soil microbial diversity patterns (Shen et al., 2020). For example, a recent study in China’s Changbai Mountains showed that the soil TC/TN ratio seems to be a key factor in shaping soil fungal distribution along small-scale elevational gradients (Ni et al., 2018). In this study, soil pH generally showed a weaker effect than the other soil variables, which can likely be attributed to the narrow pH range (8.24–8.95) and limited variation among the sampling sites (Supplementary Tables 1, 2). By contrast, the soil microbial diversity patterns were strongly affected by the soil TC/TN ratio and by TN and MAT, and their roles may also differ among the three diverse groups. Based on linear regression analysis between the environmental variables and the Chao1 index, we found that the bacterial diversity was negatively correlated with soil TC/TN ratio and positively correlated with soil TN (Tables 1, 2). In contrast with the bacteria, eukaryota diversity was also significantly negatively correlated with the soil TC/TN ratio, but significantly positively correlated with MAP and soil TN. In addition, only diversity of the archaeal community was negatively correlated with the soil TC/TN ratio. However, the relative importance of the aforementioned factors is still unclear and needs to be further studied; for example, it might be possible to test this importance using techniques such as random forest model analysis.

Clear and significant differences in soil microbial community composition along the elevational gradient were revealed by PCA, RDA, and VPA. The PCA results showed that the soil community compositions for bacteria, archaea, and eukaryota differed somewhat among the sampling locations. This also agrees with the abundance analysis (Figure 3). The microbial community compositions of samples from high- and low-elevation sites showed obvious differentiation. Furthermore, the RDA and VPA results further emphasized that a single factor could not explain the soil microbial distribution patterns (Fierer and Jackson, 2006). In this study, the combination of edaphic parameters and geographic factors explained only 4.6% of the bacterial community variation; the EC and the TC/TN ratio explained 11.9 and 6.9% of the variation, respectively, for the bacteria, leaving 95.4% of the variation unexplained. Similarly, edaphic parameters and geographic factors had a limited contributed to shaping the archaeal and eukaryota community composition (Figure 5). The combination of environmental parameters we selected can explain the soil microbial community compositions to some extent, but there is still a considerable amount of community variation that could not be explained. This may be because we did not consider several important environmental variables, such as the intensity of UV light (Jones and Lennon, 2010) and the quality and quantity of soil organic matter (Delgado-Baquerizo et al., 2017), and this may have a profound effect on the composition and distribution of the soil microbial communities. In addition, we did not account for the effects of the vegetation cover, the effects of plant species distribution along the elevational gradient, or how the microbial community varied with increasing distance from certain plant species. These factors would obviously have an important effect on microbial species diversity for rhizobia and mycorrhizal species (De Boer et al., 2006), but would likely have affected other microbes too. Thus, the roles of these factors need further study.

### More Intradomain Than Interdomain Connections Were Found for OTUs, and Most of These Connections Were Positive Links

It is clear that soil microbes do not exist in isolation, but instead coexist and together build complex ecological networks (Faust and Raes, 2012; Faust et al., 2015; Graham et al., 2016), leading to different ecologically important and complex interactions, including but not limited to mutualistic and predator–prey interactions (Mougi and Kondoh, 2012). In this study, we found that the intradomain interactions (within the bacteria, the archaea, or the eukaryota) among species formed more connections than did interdomain interactions (between any pair of these species), suggesting that intradomain OTUs can more easily form a mutualistic community, as most of these links were positive within the network. A recent study of the alpine grassland in the Eastern Tibetan Plateau also found that the positive links overwhelmingly dominated the negative links. Such positive connections among different taxa have been widely exhibited in various natural niches (Faust et al., 2015; Shi et al., 2019). It is understandable that these positive connections formed, as they could promote resistance to external environmental stress, especially in high-elevation regions with low temperatures and strong UV radiation, as in our research area. Furthermore, we found that the bacterial group formed more connections than either the archaea or the eukaryota in the Qilian Mountains, but the archaea and eukaryota may nonetheless play a significant role in constructing the microbial co-occurrence network in this area. For example, mycorrhizal symbioses by members of the eukaryota are an essential mechanism for survival of many plant species in harsh environments (De Boer et al., 2006).

## Conclusion

We found a significant linear increase in microbial diversity with increasing elevation for soil bacteria and eukaryota along the elevational gradient in alpine meadows of the Qilian Mountains, whereas archaeal diversity showed a non-significant increase. We also found that the response of the diversity of different phyla in the bacterial, archaeal, and eukaryota communities was not always consistent with the response by the overall microbial community in soils. Furthermore, we found that the soil microbial community composition was determined by the coupled effects of regional climate and local edaphic factors. The intradomain links far outweighed the interdomain links in the microbial network of our study sites, and these links were mostly positive (i.e., mutualisms). Finally, bacteria formed more connections than either archaea or eukaryota in the Qilian Mountains soils, but archaea may be more important than bacteria in constructing the microbial co-occurrence network in this region. Our findings provide insights into the formation and maintenance of soil microbial diversity along an elevational gradient and may have implications for microbial responses to climate change in alpine ecosystems.

## Data Availability Statement

The datasets presented in this study can be found in online repositories. The names of the repository/repositories and accession number(s) can be found below: https://www.ncbi.nlm.nih.gov/, SRP292149.

## Author Contributions

YDi, LW, XW, YLi, and JM: sample collection. YDi, JL, and FW: data curation. YLi: funding acquisition. YDi, JL, LW, XW, FW, WW, YLi, JZ, and YDi: methodology. YDi, XW, and YLi: project administration. YDi, JL, and YLi: writing—original draft. YDi, YLi, FW, WW, and YLi: writing—review and editing. All authors contributed to the article and approved the submitted version.

## Conflict of Interest

JZ and YD were employed by the company Shanghai Majorbio Bio-Pharm Technology Co., Ltd. The remaining authors declare that the research was conducted in the absence of any commercial or financial relationships that could be construed as a potential conflict of interest.

## Footnotes

1 http://data.cma.cn/

2 https://www.ncbi.nlm.nih.gov/sra

3 https://ccb.jhu.edu/software/FLASH/

4 https://github.com/OpenGene/fastp

5 https://drive5.com/uparse/

6 https://github.com/mothur/mothur.github.io

7 https://www.arb-silva.de/

8 https://unite.ut.ee/

9 https://www.r-project.org/

10 https://cran.r-project.org/web/packages/vegan/index.html
