# Supplementary material for: Variation in Soil Microbial Communities Along an Elevational Gradient in Alpine Meadows of the Qilian Mountains, China
Source: Front Microbiol. 2021 Jun 25;12:684386. doi: 10.3389/fmicb.2021.684386 (PMC8270674; doi:10.3389/fmicb.2021.684386)
Supplement: Supplementary file 1 [file Data_Sheet_1.docx]

**Supplementary Materials**

***Frontiers in microbiology***

**Variation in soil microbial communities along an elevational gradient in alpine meadows of the Qilian Mountains, China**

**Yulong Duan^a, b, #^, Jie Lian^a, b, #^, Lilong Wang^a, b^, Xuyang Wang****^a, b^, Yongqing Luo^a, b^, Wanfu Wang^a, c, d, e^, Fasi Wu^d, e^,** **Jianhua Zhao^f^, Yun Ding^f^, Jun Ma^g^, Yulin Li^a, b, c^, Yuqiang Li^a, b, c^** *

**^a^** *Northwest Institute of Eco-Environment and Resources, Chinese Academy of Sciences,* *Lanzhou 730000, P. R. China.*

**^b^** *Naiman Desertification Research Station, Northwest Institute of Eco-Environment and Resources, Chinese Academy of Sciences, Tongliao 028300, P. R. China.*

**^c^** *University of Chinese Academy of Sciences, Beijing 100049, P. R. China.*

**^d^** *National Research Center for Conservation of Ancient Wall Paintings and Earthen Sites, Dunhuang Academy, Dunhuang, Gansu, 736200, P. R. China.*

**^e^** *MOE Key Laboratory of Cell Activities and Stress Adaptations, School of Life Sciences, Lanzhou University, Lanzhou 730000, P.R. China.*

**^f^** *Shanghai Majorbio Bio-pharm Technology Co., Ltd, Shanghai 200120, P. R. China.*

**^g^** *Gansu Qilian Mountains National Nature Reserve Authority, Zhangye, Gansu, 734000, P. R. China.*

#Co-first author, contributed equally to this work (YL. Duan and J. Lian).

⁎Corresponding author at: Northwest Institute of Eco-Environment and Resources, Chinese Academy of Sciences, 320 Donggang West Road, Lanzhou 730000, China (YQ. Li).

*E-mail address:* [Duanyulong@nieer.ac.cn](mailto:Duanyulong@nieer.ac.cn) (YL. Duan); [Lianjie@nieer.ac.cn](mailto:Lianjie@nieer.ac.cn) (J. Lian); [Liyq@lzb.ac.cn](mailto:Liyq@lzb.ac.cn) (YQ. Li).


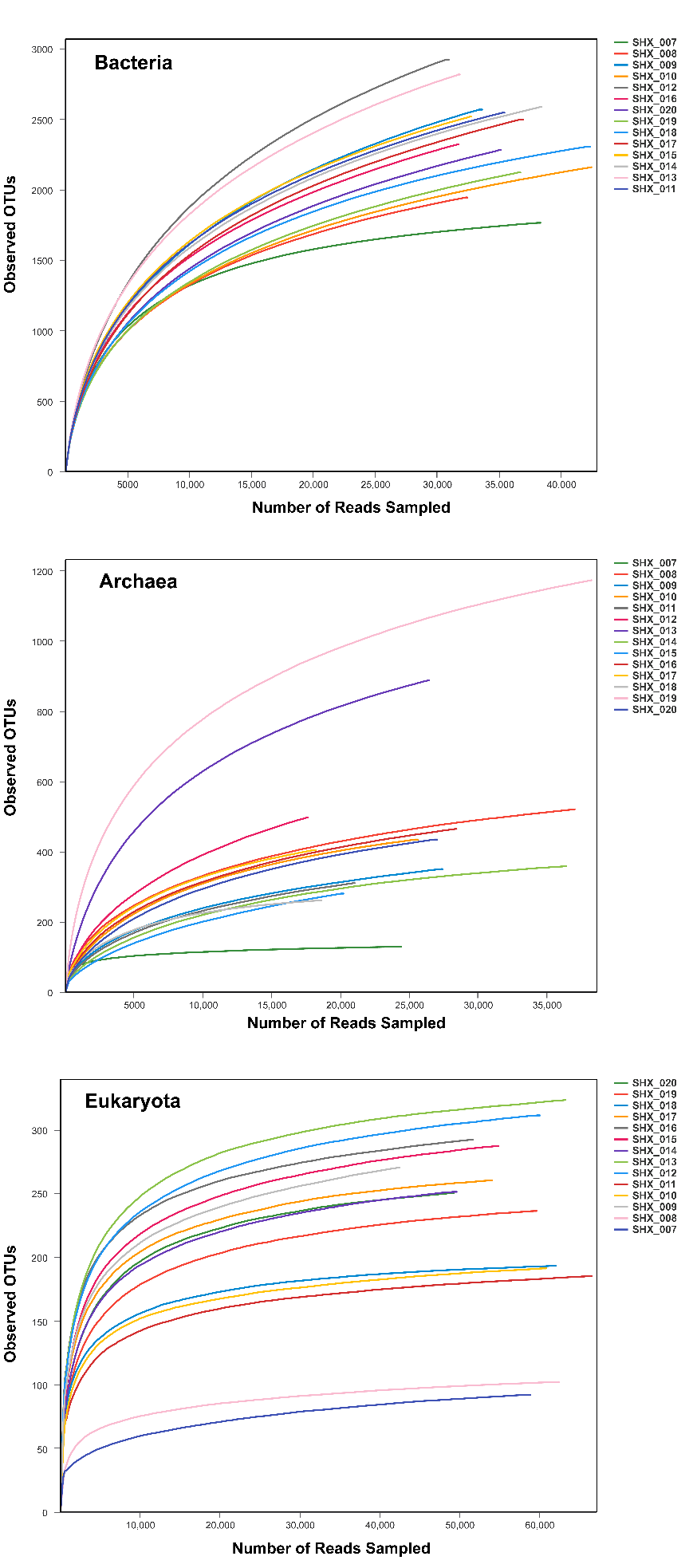


**Fig. S1. Rarefaction curves.** Rarefaction analysis of the observed operational taxonomic units (OTUs) is presented using different colors for samples from the 14 sites defined in Table S1.


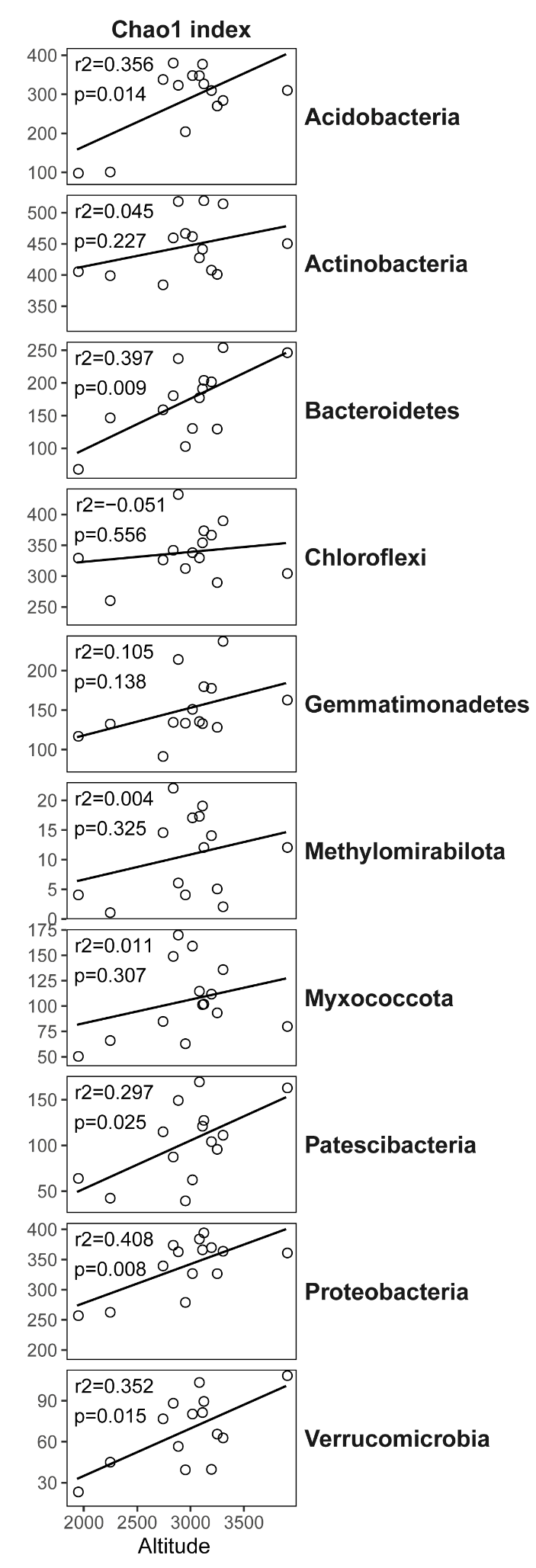


**Fig. S2. Relationships between elevation and Chao1 index for the top 10 bacterial phyla.**


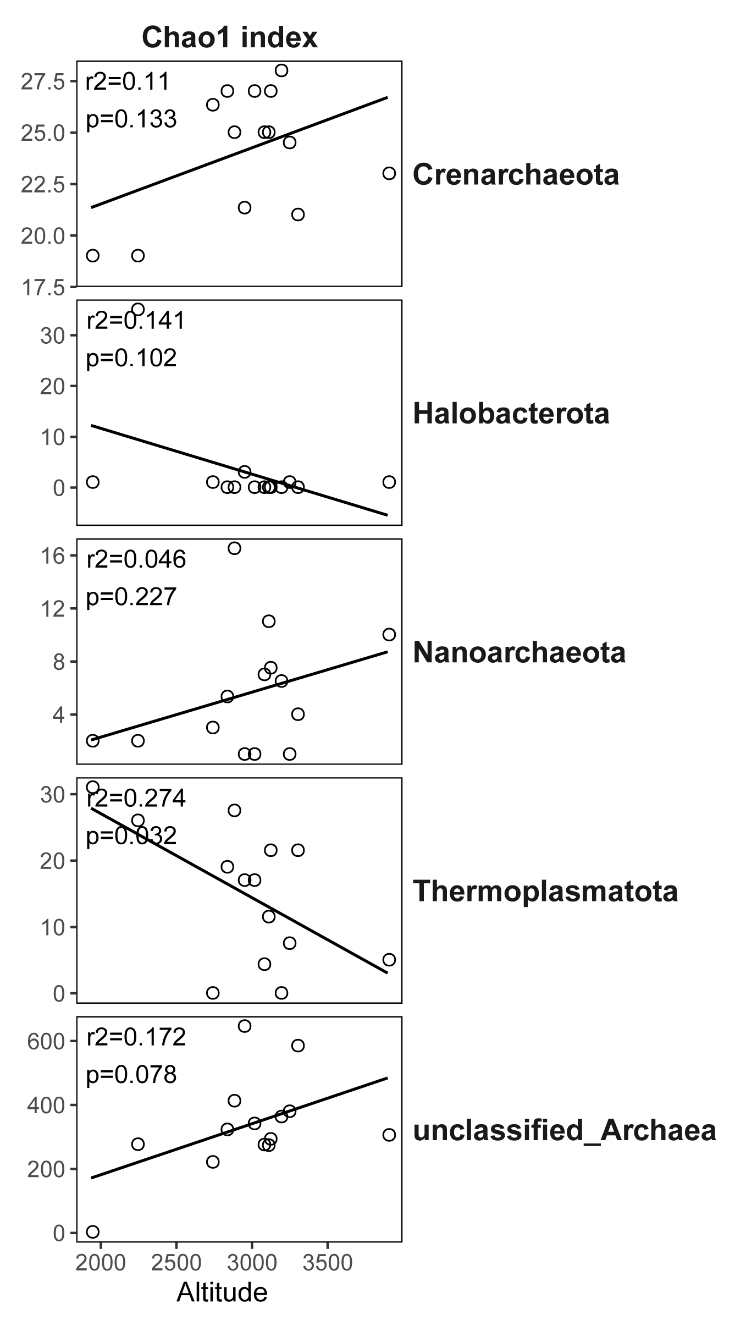


**Fig. S3. Relationships between elevation and Chao1 index for the top five archaeal phyla.**


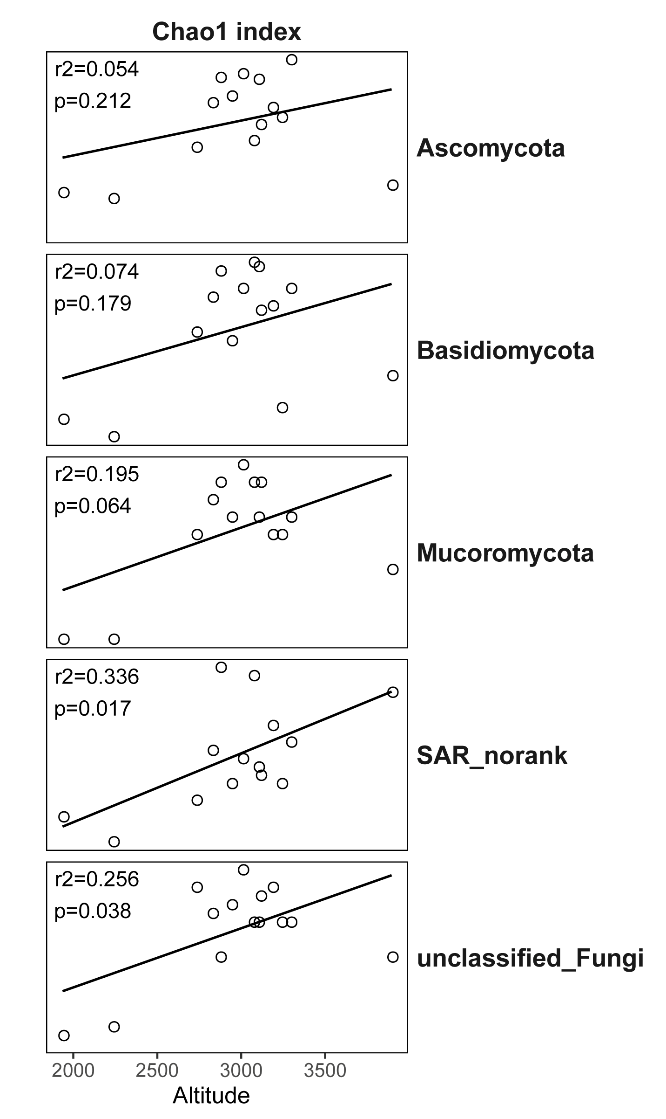


**Fig. S4. Relationships between elevation and Chao1 index for the top five fungal phyla.**

**Table S1 Locations of the sampling sites and meteorological data.**

| Sample ID | Latitude (°N) | Longitude (°E) | Elevation (m) | IM ^a^ | MAT ^a^ | MAP ^a^ | Aridity ^a^ |
| --- | --- | --- | --- | --- | --- | --- | --- |
| SHX_007 | 39.838783 | 97.930497 | 1936 | -48.10 | 4.4 | 98.1 | 5.395 |
| SHX_008 | 39.762238 | 97.788113 | 2235 | -46.90 | 3.2 | 103.0 | 4.925 |
| SHX_009 | 39.605533 | 97.702177 | 3113 | -44.34 | -3.5 | 115.5 | 15.173 |
| SHX_010 | 39.476482 | 97.68075 | 3238 | -39.37 | -4.4 | 145.3 | — |
| SHX_011 | 39.363582 | 97.651752 | 3896 | -36.11 | -7.4 | 161.9 | — |
| SHX_012 | 39.309462 | 97.880003 | 2873 | -32.71 | -1.2 | 180.8 | 2.470 |
| SHX_013 | 39.11318 | 98.176408 | 3293 | -15.58 | -3.8 | 263.7 | — |
| SHX_014 | 38.433893 | 99.578432 | 3100 | 21.10 | -4.2 | 423.6 | — |
| SHX_015 | 38.361722 | 99.72065 | 3071 | 15.41 | -4.3 | 408.2 | — |
| SHX_016 | 38.289237 | 99.851733 | 3006 | 5.80 | -1.6 | 390.3 | 1.240 |
| SHX_017 | 38.232072 | 99.995093 | 2826 | 0.40 | -1.4 | 396.1 | 1.678 |
| SHX_018 | 38.222858 | 100.104153 | 2730 | 0.13 | -0.9 | 410.1 | 1.678 |
| SHX_019 | 38.082083 | 100.365418 | 2940 | -0.03 | -1.2 | 411.3 | 1.711 |
| SHX_020 | 37.996997 | 100.762145 | 3184 | -6.08 | -7.6 | 378.0 | — |

**^a^** IM, humidity index; MAT, mean annual temperature; MAP, mean annual precipitation; aridity, annual pan evaporation divided by MAP (—, could not be calculated).

**Table S2 Description of the soil variables.**

| Sample ID | pH | Electrical conductivity | TC ^a^ | TN ^a^ | TC/TN ratio ^a^ |
| --- | --- | --- | --- | --- | --- |
| SHX_007 | 8.336 | 576 | 1.7259 | 0.0243 | 71.1618 |
| SHX_008 | 8.583 | 1095.7 | 2.1528 | 0.0508 | 42.3592 |
| SHX_009 | 8.92 | 255.17 | 1.7032 | 0.0914 | 18.6386 |
| SHX_010 | 8.246 | 211.2 | 2.1366 | 0.2038 | 10.4851 |
| SHX_011 | 8.523 | 136.6 | 5.0807 | 0.2941 | 17.2765 |
| SHX_012 | 8.236 | 1608 | 4.3976 | 0.3044 | 14.4445 |
| SHX_013 | 8.596 | 1699 | 4.7425 | 0.3450 | 13.7479 |
| SHX_014 | 8.373 | 178.16 | 5.1284 | 0.4312 | 11.8937 |
| SHX_015 | 8.286 | 168.1 | 3.0863 | 0.2337 | 13.2042 |
| SHX_016 | 8.82 | 158.87 | 3.5707 | 0.2264 | 15.7696 |
| SHX_017 | 8.95 | 152.27 | 4.4308 | 0.3859 | 11.4806 |
| SHX_018 | 8.87 | 134.33 | 2.7664 | 0.2031 | 13.6228 |
| SHX_019 | 8.463 | 124.53 | 3.7547 | 0.3097 | 12.1246 |
| SHX_020 | 8.65 | 125.4 | 1.2864 | 0.0837 | 15.3732 |

^a^ TC, total carbon; TN, total nitrogen; TC/TN, ratio of total carbon to total nitrogen.

**Table S3 Phylotype coverage and diversity estimation for the bacterial community of the samples from the MiSeq sequencing analysis.**

| Samples | Valid Sequences | Observed OTUs ^a^ | Rarefied Sequences | Mean length (bp) | Chao1 | ACE | Shannon | Simpson | Coverage |
| --- | --- | --- | --- | --- | --- | --- | --- | --- | --- |
| SHX_007 | 41091 | 1763 | 30671 | 414.5195 | 1929.457 | 1894.288 | 6.23426 | 0.005469 | 0.993493 |
| SHX_008 | 35829 | 1943 | 30671 | 416.4957 | 2458.686 | 2441.476 | 6.113681 | 0.006337 | 0.983432 |
| SHX_009 | 42477 | 2566 | 30671 | 417.3506 | 3407.014 | 3425.742 | 6.332279 | 0.005539 | 0.974885 |
| SHX_010 | 55204 | 2158 | 30671 | 417.2327 | 2761 | 2745.462 | 6.173745 | 0.005651 | 0.98576 |
| SHX_011 | 49195 | 2544 | 30671 | 418.1102 | 3309.054 | 3302.568 | 6.365693 | 0.005504 | 0.978096 |
| SHX_012 | 48859 | 2921 | 30671 | 415.9269 | 3884.241 | 3932.857 | 6.53191 | 0.0045 | 0.968081 |
| SHX_013 | 49649 | 2815 | 30671 | 415.2336 | 3704.199 | 3750.704 | 6.580665 | 0.005035 | 0.971514 |
| SHX_014 | 53402 | 2585 | 30671 | 419.2887 | 3289.049 | 3369.297 | 6.429756 | 0.004234 | 0.979769 |
| SHX_015 | 47068 | 2516 | 30671 | 418.3007 | 3260.157 | 3306.369 | 6.46473 | 0.004189 | 0.976287 |
| SHX_016 | 51519 | 2319 | 30671 | 418.8108 | 3194.955 | 3155.015 | 6.283454 | 0.005707 | 0.975888 |
| SHX_017 | 58887 | 2496 | 30671 | 419.8075 | 3312.151 | 3289.18 | 6.337829 | 0.004415 | 0.978568 |
| SHX_018 | 50657 | 2304 | 30671 | 417.5475 | 2813.511 | 2824.091 | 6.169423 | 0.006496 | 0.985845 |
| SHX_019 | 42348 | 2121 | 30671 | 416.4939 | 2815.96 | 2779.352 | 6.128198 | 0.005823 | 0.982432 |
| SHX_020 | 58163 | 2281 | 30671 | 416.1825 | 3163.964 | 3048.589 | 6.156951 | 0.006364 | 0.978973 |

**^a^** The operational taxonomic units (OTUs) were defined with a 97% similarity threshold. The variables represent the coverage percentages, richness estimators (observed OTUs, ACE, and Chao1), and diversity indices (Shannon and Simpson).

**Table S****4 Phylotype coverage and diversity estimation for the archaeal community of the samples from the MiSeq sequencing analysis**.

| Samples | Valid Sequences | Observed OTUs ^a^ | Rarefied Sequences | Mean length (bp) | Chao1 | ACE | Shannon | Simpson | Coverage |
| --- | --- | --- | --- | --- | --- | --- | --- | --- | --- |
| SHX_007 | 25673 | 129 | 17561 | 268.2028 | 144.1111 | 144.8161 | 3.528837 | 0.051013 | 0.999299 |
| SHX_008 | 44227 | 520 | 17561 | 268.8196 | 635.069 | 649.4014 | 3.26899 | 0.100098 | 0.996157 |
| SHX_009 | 34731 | 350 | 17561 | 268.6317 | 482.75 | 482.5016 | 2.734526 | 0.15612 | 0.995675 |
| SHX_010 | 29325 | 434 | 17561 | 268.2482 | 532.4375 | 547.8323 | 2.719653 | 0.167711 | 0.995042 |
| SHX_011 | 27053 | 310 | 17561 | 271.9758 | 443.4 | 448.7397 | 2.761075 | 0.12891 | 0.99446 |
| SHX_012 | 21266 | 497 | 17561 | 270.9199 | 760.6494 | 747.9292 | 3.067986 | 0.13281 | 0.988497 |
| SHX_013 | 32620 | 887 | 17561 | 269.5799 | 1123.163 | 1119.012 | 3.458886 | 0.130754 | 0.989989 |
| SHX_014 | 48771 | 358 | 17561 | 268.1954 | 413.8125 | 433.2211 | 2.812892 | 0.106031 | 0.997387 |
| SHX_015 | 28320 | 280 | 17561 | 270.5774 | 457.8 | 559.7165 | 2.672423 | 0.114704 | 0.993653 |
| SHX_016 | 36440 | 464 | 17561 | 269.354 | 607.1316 | 619.528 | 2.969591 | 0.131039 | 0.994773 |
| SHX_017 | 23299 | 404 | 17561 | 268.9356 | 494.3976 | 520.8247 | 3.40584 | 0.070644 | 0.993196 |
| SHX_018 | 24314 | 261 | 17561 | 268.6684 | 285.5 | 291.1858 | 2.702148 | 0.170499 | 0.997343 |
| SHX_019 | 48950 | 1172 | 17561 | 268.3168 | 1423.785 | 1413.141 | 4.65396 | 0.04818 | 0.992491 |
| SHX_020 | 37270 | 434 | 17561 | 269.4632 | 579.0141 | 578.0782 | 2.909557 | 0.117134 | 0.994633 |

* The operational taxonomic units (OTUs) were defined with a 97% similarity threshold. The variables represent the coverage percentages, richness estimators (observed OTUs, ACE and Chao), and diversity indices (Shannon and Simpson).

**Table S5 Phylotype coverage and diversity estimation for the eukaryota community of the samples from the MiSeq sequencing analysis**.

| Samples | Valid Sequences | Observed OTUs ^a^ | Rarefied Sequences | Mean length (bp) | Chao1 | ACE | Shannon | Simpson | Coverage |
| --- | --- | --- | --- | --- | --- | --- | --- | --- | --- |
| SHX_007 | 64293 | 92 | 42299 | 381.9836 | 113 | 115.7934 | 2.828762 | 0.099827 | 0.999642 |
| SHX_008 | 63845 | 102 | 42299 | 382.3738 | 113.6667 | 114.1652 | 1.558956 | 0.409399 | 0.99976 |
| SHX_009 | 45895 | 270 | 42299 | 382.1716 | 323.1176 | 307.2889 | 3.683863 | 0.055122 | 0.998983 |
| SHX_010 | 64673 | 191 | 42299 | 382.1498 | 200.5625 | 203.3906 | 3.149072 | 0.084523 | 0.999703 |
| SHX_011 | 72406 | 185 | 42299 | 381.5824 | 202.2727 | 198.5088 | 3.352272 | 0.065553 | 0.999699 |
| SHX_012 | 68265 | 311 | 42299 | 381.7824 | 331.3077 | 332.618 | 4.006426 | 0.037702 | 0.999447 |
| SHX_013 | 73633 | 323 | 42299 | 381.4143 | 346.25 | 340.246 | 3.994105 | 0.035133 | 0.999509 |
| SHX_014 | 54336 | 251 | 42299 | 381.5988 | 272.1364 | 271.031 | 3.54682 | 0.058513 | 0.99937 |
| SHX_015 | 60845 | 287 | 42299 | 381.5824 | 310.375 | 311.0894 | 3.507583 | 0.06632 | 0.999377 |
| SHX_016 | 57313 | 292 | 42299 | 381.9721 | 319.5556 | 313.6478 | 4.110018 | 0.028525 | 0.999379 |
| SHX_017 | 59309 | 260 | 42299 | 381.7632 | 279.1176 | 275.8496 | 3.745679 | 0.049347 | 0.999517 |
| SHX_018 | 68248 | 193 | 42299 | 381.8315 | 196.8824 | 199.246 | 3.587594 | 0.048093 | 0.999805 |
| SHX_019 | 64368 | 236 | 42299 | 381.2441 | 253.6471 | 250.7941 | 3.608871 | 0.048079 | 0.999578 |
| SHX_020 | 53384 | 250 | 42299 | 381.5478 | 270.6471 | 265.8094 | 3.446732 | 0.064683 | 0.999448 |

^a^ The operational taxonomic units (OTUs) were defined with a 97% similarity threshold. The variables represent the coverage percentages, richness estimators (Observed OTUs, ACE and Chao), and diversity indices (Shannon and Simpson).

**Table S11 Numbers of links between the bacteria, archaea, and eukaryota in the soil microbial network.**

|  | ***No. of nodes*** | ***Correlation type*** | ***Bacteria*** | ***Archaea*** | ***Eukaryota*** |
| --- | --- | --- | --- | --- | --- |
| **Bacteria** | 1,181 | Positive | 5366 (76.19%) | 2068 (94.51%) | 213 (87.65%) |
|  |  |  |  |  |  |
|  |  | Negative | 1677 (23.81%) | 120 (5.49%) | 19 (12.35%) |
|  |  |  |  |  |  |
| **Archaea** | 354 | Positive | - | 1427 (99.86%) | 252 (100%) |
|  |  |  |  |  |  |
|  |  | Negative | - | 2 (0.14%) | 0 (0%) |
|  |  |  |  |  |  |
| **Eukaryota** | 166 | Positive | - | - | 139 (100%) |
|  |  |  |  |  |  |
|  |  | Negative | - | - | 0 (0%) |
|  |  |  |  |  |  |
